# Chronic Headache Attributed to Vertebrobasilar Insufficiency

**DOI:** 10.3390/diagnostics12092038

**Published:** 2022-08-23

**Authors:** Sang Woo Ha, Young Seo Kim, Eun Joo Yoon, Hyun Goo Kang

**Affiliations:** 1 Department of Neurosurgery, Chosun University School of Medicine, Gwangju 61453, Korea; 2 Department of Neurology, Wonkwang University School of Medicine, Iksan 54538, Korea; 3 Department of Radiology, Chosun University School of Medicine, Gwangju 61453, Korea; 4 Department of Neurology and Research, Institute of Clinical Medicine of Jeonbuk National University-Biomedical Research Institute of Jeonbuk National University Hospital, Jeonju 54907, Korea

**Keywords:** aneurysm, headache, stent, vertebral artery, vertebrobasilar insufficiency

## Abstract

Vertebrobasilar insufficiency, a condition characterized by poor blood flow to the posterior portion of the brain, can cause headaches. However, the exact underlying mechanism is not yet fully understood. The patient enrolled in our study reported experiencing intermittent headaches radiating from the left shoulder, similar to chronic tension-type headaches. His aggravated headache and severe left vertebral artery stenosis were detected by brain computed tomography angiography. Stent insertion successfully expanded the patient’s narrowed left vertebral artery orifice. Subsequently, the patient’s headaches improved without recurrence during the one-year follow-up period. In summary, chronic headaches attributed to vertebrobasilar insufficiency in this study, improved after stent insertion to reverse severe left vertebral artery stenosis.

A 45-year-old male had suffered intermittent compressive left occipital headache radiating from the left shoulder for 5 years (10–15 days per month). His headache had worsened for a month (7/10 on the Visual Analog Scale). Brain computed tomography angiography showed severe stenosis at the orifice of the left vertebral artery (VA). A stent was inserted and the location of the balloon-mounted stent (Biotronik, Pro-kinteic Energy 5.0 × 13 mm) was confirmed under fluoroscopic guidance (Figure 1a,b). Due to the atheroma of the VA ostium, the balloon-mounted stent (arrow) distally migrated during inflation because of the hardening plaque (Figure 1c,d). It can be observed that the atheroma moved down from the VA origin as a result (Red block). An expansion of the narrowed left VA orifice (balloon angioplasty using Submarine 6–20) was confirmed, and the stent was positioned properly and VA flow improved (Figure 1e,f). His headache improved in a few days without recurrence during the one-year follow-up. Vertebrobasilar insufficiency can cause headaches, although the mechanism is not understood. Bow hunter’s syndrome, which occurs due to the rotational compression of the VA, accompanies headaches in approximately 7–9% of cases [1,2]. Few reports also showed that headache often occurs in lateral medullary ischemia due to an atherothrombotic cause (52–73%) [3,4]. The chronic headache, reported and attributed to vertebrobasilar insufficiency in this study, rapidly improved after stent insertion for the left VA severe stenosis.

**Figure 1:**
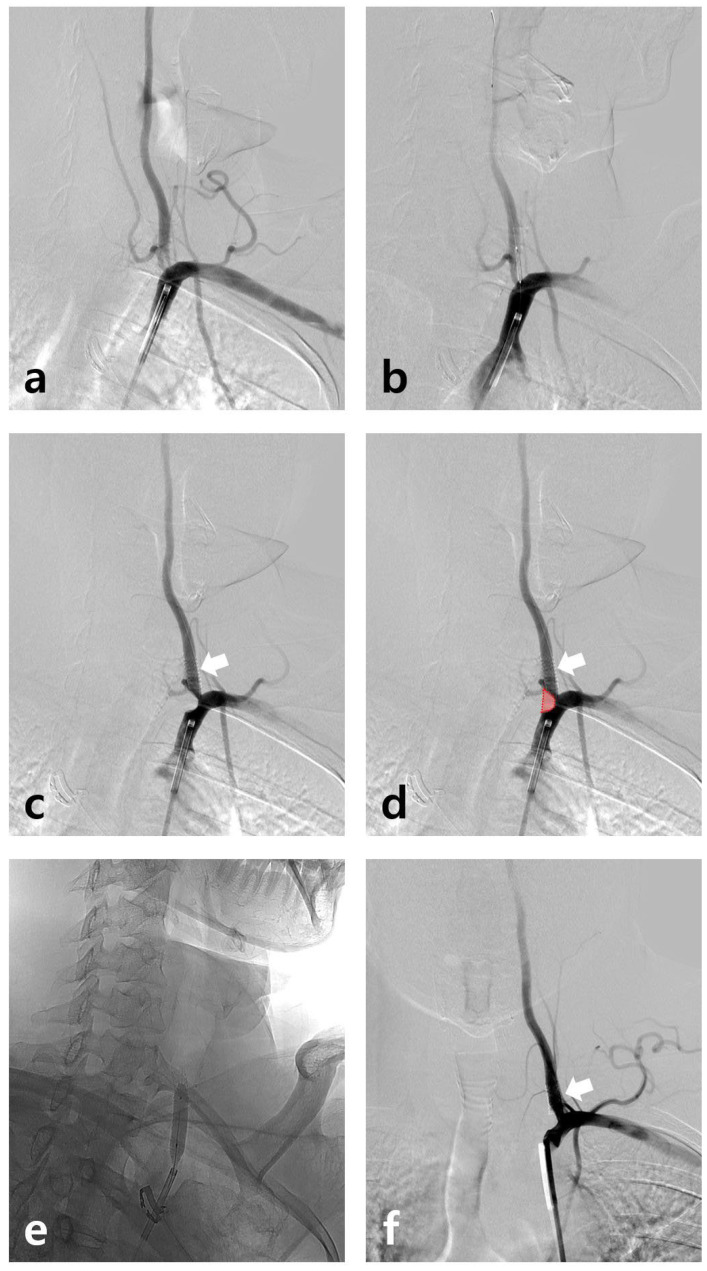
(**a**) Stenosis from the left VA ostium confirmed on subclavian artery angiography. (**b**) The location of the balloon-mounted stent (Biotronik, Pro-kinteic Energy 5.0 × 13 mm) confirmed under fluoroscopic guidance. (**c**,**d**) Due to the atheroma of the VA ostium, the balloon-mounted stent (arrow) distally migrated during inflation. It can be observed that the atheroma moved down from the VA origin as a result (red block). (**e**) Additional balloon angioplasty using Submarine 6–20 was conducted in the ostium. (**f**) The stent is well-maintained (arrow) and VA flow improved.

## Data Availability

Data available on request due to restrictions eg privacy or ethical.
